# The subgingival microbiomes in periodontitis and health of individuals with rheumatoid arthritis and at risk of developing rheumatoid arthritis

**DOI:** 10.1080/20002297.2017.1325216

**Published:** 2017-05-30

**Authors:** Z. Cheng, T. Do, K. Mankia, J.L. Meade, L. Hunt, J. Nam, A. Tugnait, A. Speirs, V. Clerehugh, P. Emery, D. Devine

**Affiliations:** ^a^ Division of Oral Biology, University of Leeds, School of Dentistry, UK.; ^b^ Leeds Musculoskeletal Biomedical Research Centre, University of Leeds, School of Medicine, UK; ^c^ Division of Restorative Dentistry, University of Leeds, School of Dentistry, UK.; ^d^ Leeds Dental Institute, Leeds Teaching Hospitals Trust, UK

## Abstract

Serum anti-citrullinated protein antibodies (ACPAs), present in 70% of people with rheumatoid arthritis (RA), can be detected ≤10years before the onset of clinical disease. RA and periodontitis are epidemiologically associated and we have reported a high incidence of periodontitis in people who are ACPA+ and at risk of RA. Periodontal bacteria may contribute by multiple routes to the generation of RA-autoantibodies. This study aims to characterise the subgingival microbiomes from periodontitis and health in individuals with/without RA and at risk of RA.

Forty-five ACPA+ no RA (RA-at-risk; RAR), 31 healthy controls (HC) and 30 ACPA+ RA patients (RA) underwent a periodontal examination. DNA from subgingival plaque from healthy and deep pocket sites were paired-end sequenced using the Illumina HiSeq3000 and data analysed using MG-RAST + DESeq.

Metagenomes in RA samples had high proportions of Actinobacteria; RAR microbiomes contained higher proportions of Bacteroidetes than HC. The relative abundance of *P. gingivalis* was high in periodontitis and RAR; *Aggregatibacter actinomycetemcomitans* was detected with similar frequency in each group. Other bacteria implicated in periodontitis and/or autoantibody generation (*Filifactor alocis, Prevotella* spp, *Leptotrichia* spp.) were detected. Analyses are on-going to elucidate the diversity and functional potential of the subgingival microbiome associated with RA.

